# Beyond clinical criteria: emerging biomarkers and advanced imaging in the diagnosis, monitoring and treatment of vasculitides

**DOI:** 10.3389/fmed.2026.1865061

**Published:** 2026-06-18

**Authors:** Matteo Colina

**Affiliations:** Rheumatology Service, Section of Internal Medicine, Department of Medicine and Oncology, Ospedale Santa Maria della Scaletta, Imola, Italy

**Keywords:** ANCA, biomarkers, disease activity, giant cell arteritis, imaging, PET/CT, relapse prediction, Takayasu arteritis

## Abstract

Vasculitides represent a heterogeneous group of immune-mediated diseases characterized by inflammation of blood vessel walls, leading to variable degrees of ischemic injury across multiple organ systems. Despite significant advances in classification—notably the 2022 ACR/EULAR criteria—the clinical management of vasculitis remains challenging, particularly with regard to accurate diagnosis, assessment of disease activity, prediction of relapse, and monitoring of treatment response. Traditional clinical and laboratory markers, including erythrocyte sedimentation rate (ESR) and C-reactive protein (CRP), lack specificity and are often insufficient for guiding individualized therapeutic decisions. In recent years, a new generation of biomarkers has emerged, encompassing serological proteins, urinary markers, cytokines, endothelial activation molecules, and exploratory multi-omic signatures. In parallel, advanced imaging modalities—including 18F-fluorodeoxyglucose positron emission tomography/computed tomography (18F-FDG PET/CT), high-resolution vessel wall magnetic resonance imaging (VW-MRI), vascular ultrasound, and magnetic resonance angiography (MRA)—have substantially expanded our ability to visualize vascular inflammation with unprecedented anatomical and functional detail. This narrative review synthesizes current evidence on the most relevant biomarkers and imaging techniques across the principal vasculitic syndromes, including anti-neutrophil cytoplasmic antibody (ANCA)-associated vasculitides (AAV), giant cell arteritis (GCA), Takayasu arteritis (TAK), and other major forms. We discuss the diagnostic and prognostic value of each approach, explore the emerging concept of integrated biomarker-imaging strategies, and identify key unmet needs and future research directions in the field.

## Introduction

The vasculitides encompass a spectrum of rare diseases unified by the pathological hallmark of blood vessel wall inflammation. Depending on the caliber of the affected vessels and the underlying immunological mechanisms, vasculitis can manifest as life-threatening organ failure—including rapidly progressive glomerulonephritis, pulmonary hemorrhage, aortic aneurysm, or stroke—or as insidious, smoldering disease that damages end organs over years (1). The classification of vasculitis has historically relied on clinicopathological criteria, but the landmark 2022 ACR/EULAR classification criteria have introduced a more rigorous, validated framework based on clinical, serological, and histological data (2–4).

Notwithstanding this progress, the clinical management of vasculitis faces persistent challenges. First, diagnosis is often delayed because early disease presents with non-specific systemic features, and definitive histological confirmation is not always feasible (5). Second, assessment of disease activity is hampered by the unreliability of non-specific inflammatory markers such as ESR and CRP, which can be elevated in infection, malignancy, and other comorbidities (6, 7). Third, predicting relapse—one of the most clinically consequential events in vasculitis management—remains an unsolved problem, often leading to either overtreatment with attendant toxicity or undertreatment with irreversible organ damage (8).

Against this backdrop, two parallel scientific advances are reshaping the landscape of vasculitis care. On one side, an expanding portfolio of emerging biomarkers—including disease-specific autoantibodies, endothelial activation markers, cytokines, and urinary proteins—promises greater diagnostic specificity and more nuanced monitoring of disease trajectory. On the other, a new generation of imaging modalities offers the ability to visualize vascular wall inflammation directly, detect subclinical vascular involvement, and quantify disease extent in ways that were previously impossible.

This narrative review aims to provide a clinically oriented synthesis of the current evidence on emerging biomarkers and advanced imaging techniques across the major vasculitic syndromes. We focus on the practical implications for diagnosis, disease activity assessment, relapse prediction, and treatment monitoring. The review is structured around the principal vasculitis categories, followed by a discussion of integrated approaches and future perspectives.

The present narrative review focuses primarily on ANCA-associated vasculitis (AAV), giant cell arteritis (GCA), Takayasu arteritis (TAK), and Behçet's disease, which represent the vasculitic syndromes with the most developed biomarker and imaging literature. Other vasculitides are discussed in dedicated subsections where relevant evidence exists. Biomarkers and imaging modalities were selected based on their clinical relevance, availability of published evidence, and potential for integration into clinical practice, prioritizing systematic reviews, meta-analyses, and large cohort studies where available. As inherent to the narrative review format, no formal inclusion or exclusion criteria were applied, and this should be considered a limitation when interpreting the breadth and balance of the evidence presented.

Throughout this review, a distinction is made between biomarkers and imaging modalities that have achieved clinical validation and are currently recommended in international guidelines, and those that remain investigational—showing proof-of-concept or early-phase evidence but not yet suitable for routine clinical application. This distinction is intended to guide clinicians in interpreting the evidence and translating it into practice.

## Classification of vasculitides: a framework for biomarkers and imaging research

The Chapel Hill Consensus Conference (CHCC) nomenclature, last revised in 2012, classifies vasculitides primarily according to vessel caliber: large-vessel vasculitis (LVV), including GCA and TAK; medium-vessel vasculitis (MVV), including polyarteritis nodosa (PAN) and Kawasaki disease; and small-vessel vasculitis (SVV), subdivided into ANCA-associated vasculitis (AAV)—comprising granulomatosis with polyangiitis (GPA), microscopic polyangiitis (MPA), and eosinophilic granulomatosis with polyangiitis (EGPA)—and immune complex vasculitis, including IgA vasculitis and cryoglobulinemic vasculitis (1).

The 2022 ACR/EULAR classification criteria represent a major refinement, incorporating ANCA serology, imaging, and histology into validated scoring systems for GCA, TAK, GPA, MPA and EGPA (2–4, 9, 10). Importantly, these criteria underscore the central role of biomarkers (particularly ANCA specificity) and imaging (particularly PET/CT and vascular ultrasound in LVV) as classification variables, foreshadowing their growing importance in routine clinical decision-making.

From a biomarker and imaging research perspective, this classification provides a useful scaffold: large-vessel vasculitides require imaging modalities capable of evaluating the aorta and its branches over long anatomical distances, and biomarkers that capture aortic wall inflammation and remodeling; small-vessel AAV requires biomarkers with high specificity for autoimmune mechanisms and imaging focused on organ-specific damage; medium-vessel vasculitis presents unique challenges due to the patchy distribution of lesions and the relative paucity of validated biomarkers.

## Emerging biomarkers in vasculitis

### ANCA and their subtypes: form diagnostic tool to disease monitor

Anti-neutrophil cytoplasmic antibodies (ANCA) directed against proteinase 3 (PR3-ANCA) or myeloperoxidase (MPO-ANCA) remain the cornerstone serological markers in AAV diagnosis and monitoring (8). The 2022 EULAR recommendations affirm that positive ANCA testing by ELISA—particularly PR3-ANCA in GPA and MPO-ANCA in MPA—carries high diagnostic specificity (exceeding 90% in high-pretest-probability populations) and should be incorporated into clinical decision algorithms before proceeding to tissue biopsy (11).

Beyond their diagnostic utility, ANCA titers have been extensively studied as predictors of relapse. Longitudinal cohort data, including analyses from the European Vasculitis Study Group (EUVAS), have demonstrated that persistent or rising PR3-ANCA titers during remission are associated with a significantly increased risk of relapse in GPA, with hazard ratio ranging from approximately 3–11 depending on renal involvement (12, 13). This relationship is less consistent for MPO-ANCA, possibly reflecting the heterogeneous mechanisms of MPO-ANCA-associated vasculitis, in which fibrotic end-organ damage can occur independently of ongoing immunological activity (8, 12, 13). Importantly, a recent study by Bossan et al. (14) demonstrated that baseline MPO-ANCA titers did not predict relapse; however, their elevation during follow-up was significantly associated with clinical deterioration, suggesting that serial monitoring of MPO-ANCA rather than baseline values is of greater clinical utility in MPO-AAV.

More recently, the concept of “ANCA-guided” therapy—modulating immunosuppressive treatment based on ANCA kinetics rather than fixed schedules—has gained traction. The MAINRITSAN2 trial explored tailored vs. fixed rituximab retreatment schedules in ANCA vasculitis, demonstrating that a biomarker-driven approach could reduce cumulative drug exposure without increasing relapse risk (15). These findings reinforce the concept of ANCA as a dynamic therapeutic biomarker rather than merely a diagnostic one.

### Interleukin-6 and the JAK-STAT pathway: from pathogenesis to clinical monitoring

Interleukin-6 (IL-6) occupies a pivotal role in the pathogenesis of large-vessel vasculitides, particularly GCA. Serum IL-6 levels are elevated in active GCA and decrease rapidly following glucocorticoid initiation, often preceding the normalization of ESR and CRP (16, 17). This kinetic advantage makes IL-6 a more sensitive early marker of treatment response compared to traditional acute-phase reactants.

The therapeutic relevance of the IL-6 pathway is underscored by the landmark GiACTA trial, which demonstrated the efficacy of the anti-IL-6 receptor antibody tocilizumab in achieving sustained glucocorticoid-free remission in GCA (18). Importantly, in patients receiving tocilizumab, serum IL-6 levels paradoxically increase due to receptor blockade and reduced clearance, rendering this marker unreliable as a disease activity monitor during tocilizumab therapy (19). Instead, soluble IL-6 receptor (sIL-6R) and downstream signaling molecules such as serum amyloid A (SAA) and fibrinogen have been proposed as alternative monitoring tools in this setting (18, 20).

In Takayasu arteritis, IL-6 has similarly been identified as a key driver of inflammation and a potential biomarker of activity (21), though its relationship to imaging-defined disease progression is less well-established (22). Emerging data suggest that persistently elevated IL-6 levels may be associated with progressive vascular remodeling and new stenotic lesions on serial imaging, even in patients who appear clinically quiescent—a phenomenon sometimes referred to as “imaging-clinical discordance” (22, 23).

### Pentraxin-3 (PTX-3): a vascular specific acute phase reactant

Pentraxin-3 (PTX3) is a long-chain pentraxin produced locally by vascular endothelial cells, smooth muscle cells, and macrophages in response to inflammatory stimuli, in contrast to the hepatic production of CRP (24, 25). This local origin makes PTX3 a more specific marker of vascular inflammation than classical systemic acute-phase reactants (25, 26).

In GCA, PTX3 levels have been shown to correlate with disease activity and to decrease following glucocorticoid therapy, with some studies reporting superior specificity for vascular inflammation compared to CRP (26, 27). In Takayasu arteritis, PTX3 has been proposed as a marker of active vessel wall inflammation, with levels correlating with the degree of arterial involvement detected on imaging (26). Crucially, PTX3 may retain diagnostic utility even when traditional inflammatory markers are normal—a scenario encountered in a subset of patients with clinically quiescent but imaging-active disease (26). Several studies have evaluated PTX3 as part of a multimarker approach for LVV disease activity assessment, though prospective validation in larger cohorts remains an unmet need.

Notably, a systematic review and meta-analysis by Wen et al. demonstrated that PTX3 is more accurate than C-reactive protein for assessing disease activity in Takayasu arteritis, supporting its superiority over conventional acute-phase reactants as a monitoring biomarker (28).

### Endothelial activation markers: VEGF, angiopoietin-2 and von Willebrand factor

Vascular endothelial growth factor (VEGF), angiopoietin-2 (Ang-2), and von Willebrand factor (vWF) are released by activated endothelial cells and reflect the degree of vascular injury and remodeling occurring in vasculitis (17, 27, 29). Elevated serum VEGF levels have been documented in active GCA and TAK, correlating with the extent of new vessel formation and collateral circulation (27, 30). In the context of ischemic complications—such as aortic aneurysm formation in GCA or renovascular hypertension in TAK—serial VEGF measurements may provide indirect evidence of ongoing vascular stress.

Angiopoietin-2, a destabilizer of endothelial tight junctions, is elevated in vasculitis flares and has been proposed as a marker of endothelial injury and ongoing vascular remodeling (29). Several studies have proposed Ang-2 as part of a composite endothelial damage score alongside vWF and soluble E-selectin, though these approaches remain investigational (29). The practical clinical use of these markers is currently limited by the lack of standardized assays and reference intervals across different patient populations (22).

### Novel serological biomarkers: galectin-3, BAFF and soluble molecule

**Figure 1:**
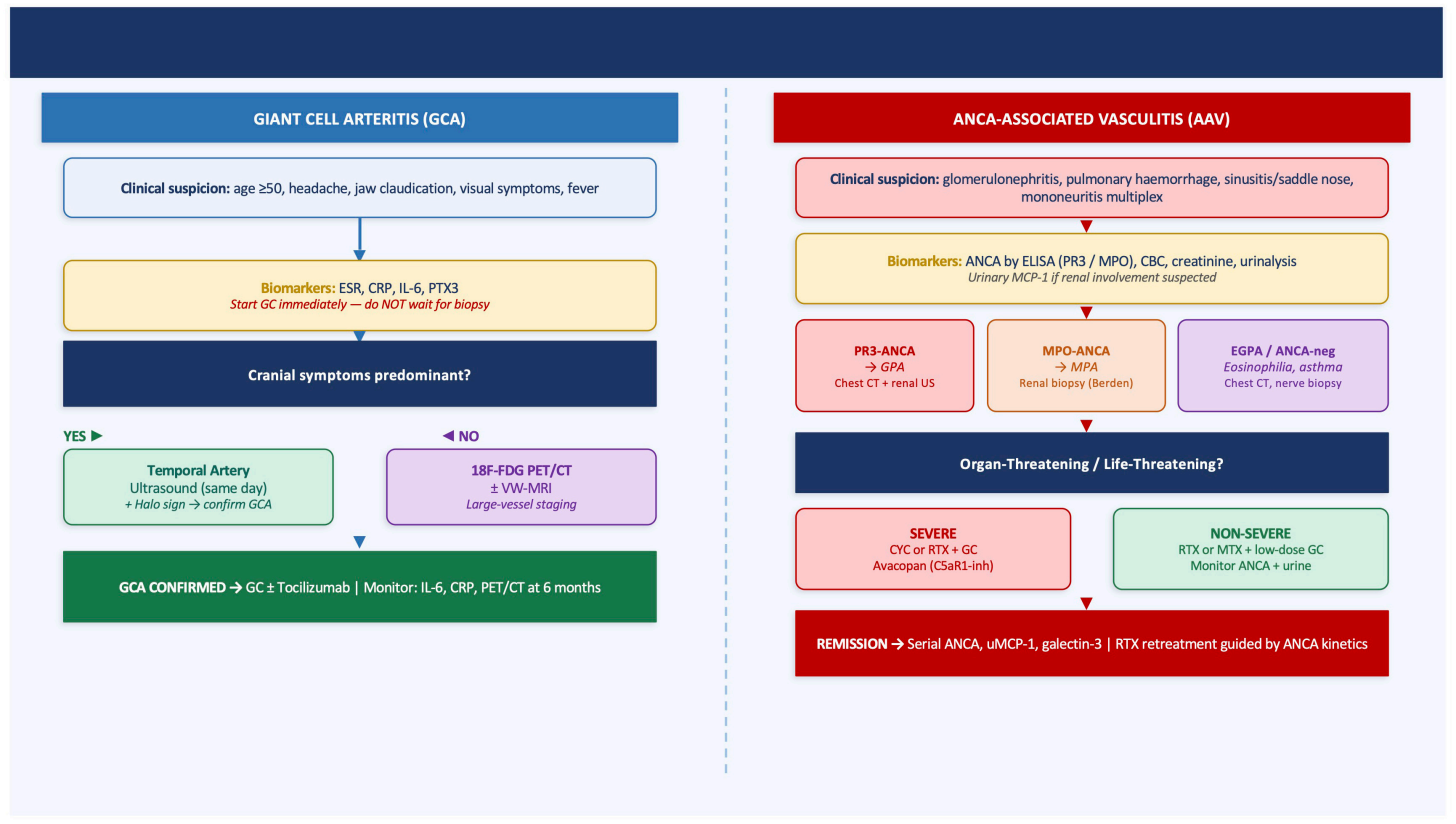
Diagnostic and monitoring algorithm for vasculitis integrating biomarkers and advanced imaging. The flowchart illustrates a stepwise clinical approach to vasculitis diagnosis and follow-up. Initial evaluation incorporates disease-specific serological biomarkers (ANCA, IL-6, complement) alongside first-line imaging modalities selected according to suspected vessel caliber. Subsequent monitoring integrates emerging biomarkers and imaging findings to guide treatment decisions, relapse assessment, and tapering strategies. AAV, ANCA-associated vasculitis; GCA, giant cell arteritis; TAK, Takayasu arteritis; PET/CT, positron emission tomography/computed tomography; VW-MRI, vessel wall magnetic resonance imaging; GC, glucocorticoids; RTX, rituximab; CYC, cyclophosphamide; uMCP-1, urinary MCP-1; PXT3, pentraxin-3.

Galectin-3 and galectin-9 have been proposed as potential biomarkers of disease activity and fibrotic progression in AAV (31). Elevated galectin-3 levels correlate with renal fibrosis and impaired glomerular filtration rate, and preliminary data in AAV suggest a potential role as a biomarker of chronic renal damage, though prospective validation in larger AAV-specific cohorts is lacking (31, 32). B-cell activating factor (BAFF, also known as BLyS) is elevated in AAV and reflects the degree of B-cell hyperactivation, which drives ANCA production and perpetuates the autoimmune cycle (17). BAFF levels have been proposed as predictors of rituximab response and as markers of B-cell reconstitution after rituximab-induced depletion—a potential guide for retreatment timing (33). The therapeutic targeting of BAFF in AAV is currently under investigation in clinical trials (Figure 1).

Other emerging candidates include soluble CD163 (a macrophage activation marker elevated in AAV flares) (34), serum calprotectin (reflecting neutrophil activation) (35, 36), and plasma cell-free DNA (cfDNA) as a emerging marker of NETs-associated cell death and tissue damage in AAV, though its correlation with disease activity remains inconsistent across studies (37). More recent evidence has further expanded the role of calprotectin across vasculitic syndromes. S100 proteins including calprotectin have been implicated in the pathogenesis of IgA vasculitis and other systemic vasculitides, with emerging data supporting their utility as biomarkers of disease activity and therapeutic response (38, 39). The scope of clinical application of serum calprotectin as a monitoring biomarker in large vessel vasculitis has also been systematically reviewed (40, 41). The clinical translation of these markers will require well-powered prospective validation studies with standardized assay methodologies.

### Urinary biomarkers in ANCA-associated vasculitis with renal involvement

Renal involvement is a defining and prognostically critical feature of AAV, occurring in up to 80% of patients with GPA and MPA (8). Current assessment of renal activity relies heavily on urinary sediment, proteinuria quantification, and serum creatinine—markers that reflect established injury rather than active inflammation (8, 11). In this context, urinary biomarkers capable of detecting active glomerulonephritis non-invasively represent an important clinical need.

Urinary monocyte chemoattractant protein-1 (MCP-1/CCL2) has emerged as one of the most promising urinary biomarkers in ANCA vasculitis nephritis (42, 43). Elevated urinary MCP-1 levels reflect active glomerular macrophage infiltration and correlate with the degree of active lesions on renal biopsy according to the Berden classification (42, 44). Several prospective studies have demonstrated that urinary MCP-1 normalizes during effective immunosuppression and rises again during renal relapse, often preceding clinical deterioration by weeks (42, 43).

Additional urinary candidates include CD163 (a marker of macrophage polarization) (34), urinary TNF-like weak inducer of apoptosis (TWEAK) (45), and kidney injury molecule-1 (KIM-1) (46). Although none has yet achieved clinical implementation, ongoing biomarker discovery studies combining urinary proteomics and metabolomics are expected to define more refined signatures of active renal vasculitis in the coming years.

### Multi-omic approaches: the future of vasculitic biomarkers discoveries

The convergence of genomics, transcriptomics, proteomics, and metabolomics—collectively referred to as multi-omics—is opening new frontiers in vasculitis biomarker research. Transcriptomic profiling of peripheral blood in AAV has identified type I interferon signatures and neutrophil extracellular trap (NET) gene expression modules that correlate with disease activity and may precede clinical relapse (47, 48). In GCA, transcriptomic profiling of temporal artery biopsies and single-cell analyses of circulating immune cells has revealed distinct macrophage and T-cell polarization states associated with different disease phenotypes and therapeutic responses (49, 50).

Plasma proteomic profiling using proximity extension assay (PEA) technology has identified multiprotein panels with superior discrimination of active vs. remission states compared to individual markers (51, 52). Metabolomic studies have revealed perturbations in amino acid and lipid metabolism in active vasculitis, with preliminary data suggesting correlation with disease activity. While these technologies are not yet clinically deployable, they represent the foundation upon which next-generation diagnostic and monitoring tools will be built.

Table 1 summarizes the principal emerging biomarkers across the major vasculitic syndromes, including Behcet's disease (BD), with an indication of their clinical roles and current level of evidence.

**Table 1:** Principal emerging biomarkers in vasculitis: clinical role and level of evidence.

Vasculitis	Biomarker	Clinical role	Level of evidence
AAV (GPA, MPA, EGPA)	ANCA (PR3/MPO)	Diagnosis, relapse prediction, treatment monitoring	High (LoE 1b)
GPA	PR3-ANCA	Correlation with disease activity; relapse predictor	High (LoE 1b)
MPA	MPO-ANCA	Diagnosis; marker of chronic renal damage	High (LoE 1b)
GCA/Takayasu	IL-6	Disease activity; tocilizumab response monitoring	Moderate (LoE 2a)
GCA/Takayasu	Pentraxin-3 (PTX3)	Vascular-specific inflammation; active wall disease	Moderate (LoE 2b)
AAV with renal involvement	Urinary MCP-1 (CCL2)	Non-invasive marker of active glomerulonephritis	Moderate (LoE 2b)
GCA/AAV	VEGF	Neoangiogenesis; disease progression	Low (LoE 3)
AAV	Galectin-3	Renal fibrosis; chronic organ damage	Low (LoE 3)
AAV	BAFF/BLyS	B-cell hyperactivation; relapse risk; RTX retreatment timing	Low (LoE 3)
GCA/Takayasu	Angiopoietin-2	Endothelial dysfunction; aortic complications	Low (LoE 3)
Behcet's disease	IL-17, IL-18, sVCAM-1	Mucocutaneous and vascular activity	Low (LoE 3)
Behcet's disease	Neutrophil-to-lymphocyte ratio (NLR)	Simple marker of systemic inflammation and flare	Low (LoE 3)

AAV, ANCA-associated vasculitis; GCA, giant cell arteritis; GPA, granulomatosis with polyangitis; MPA, microscopic polyangitis; TAK, Takayasu arteritis; NLR, neutrophil-to-lymphocyte ratio; RTX, rituximab; LoE, level of evidence.

## Advanced imaging in vasculitis

### 18 FDG-PET/CT: functional imaging of vascular inflammation

18F-Fluorodeoxyglucose positron emission tomography combined with computed tomography (18F-FDG PET/CT) has emerged as a transformative tool in the diagnosis and monitoring of large-vessel vasculitis. The technique exploits the avid glucose uptake of metabolically active inflammatory cells—particularly macrophages and activated T cells—within the vessel wall, producing characteristic patterns of mural FDG uptake that can be quantified using standardized uptake values (SUVmax) or visual scoring systems.

In GCA, PET/CT demonstrates linear, smooth, and bilaterally symmetric FDG uptake along the aorta and its major branches, a pattern distinct from the focal and irregular uptake associated with atherosclerosis (53). Meta-analyses have reported pooled sensitivities of 80%−90% and specificities of 89%−98% for PET/CT in active GCA, with performance highest when imaging is performed before or within days of glucocorticoid initiation (54). The technique is particularly valuable for detecting extra-cranial LVV in patients with atypical presentations or negative temporal artery biopsy (TAB), where it can reveal aortic and subclavian involvement not evident on clinical examination (53).

**Figure 2:**
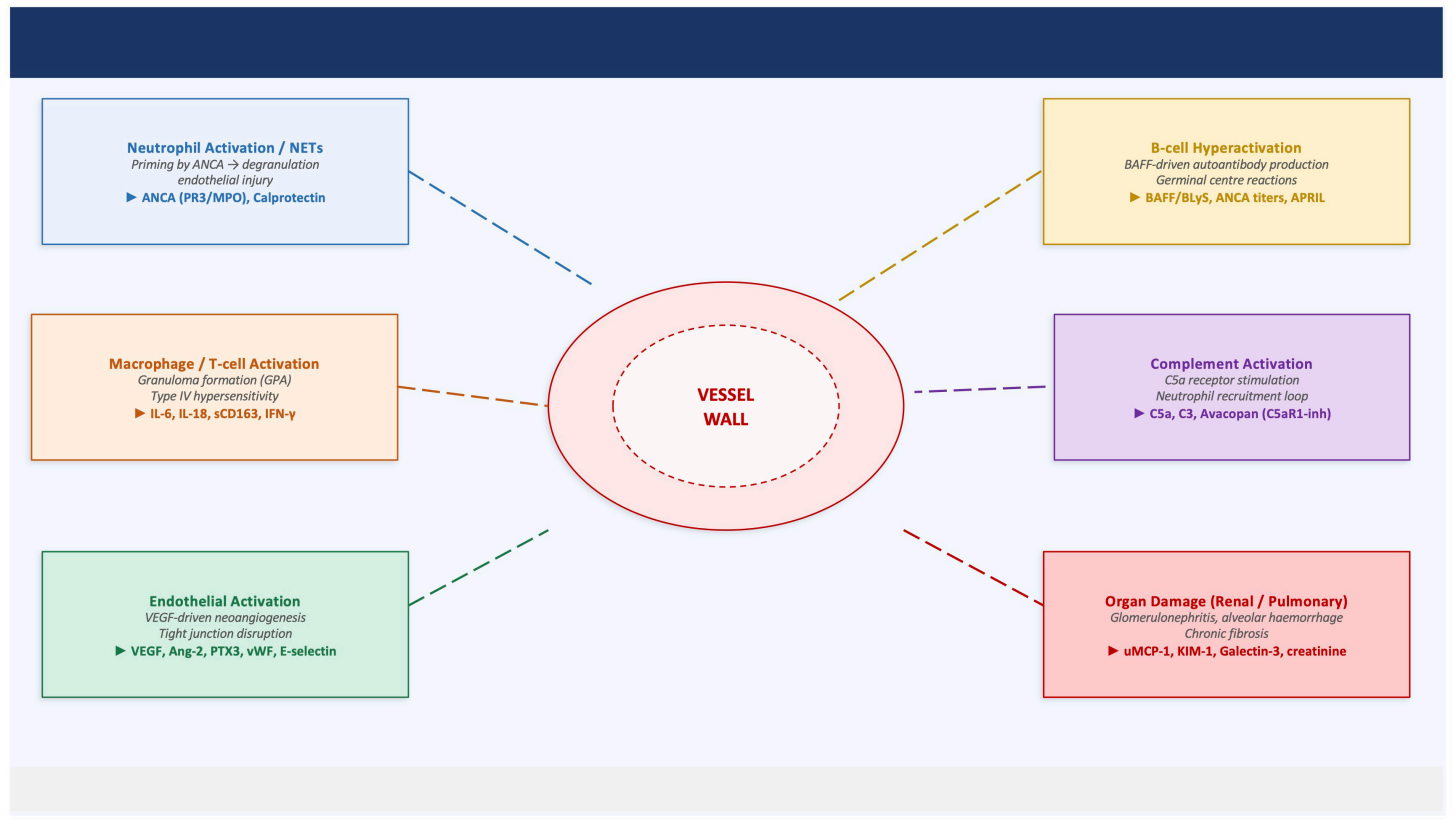
Immunopathogenesis of vasculitis and corresponding biomarker targets. Each node represents a distinct pathogenetic mechanism contributing to vessel wall inflammation. Dashed lines indicate mechanistic connections to the vessel wall. Arrows indicate corresponding clinical biomarkers under investigation or in clinical use. ANCA, antineutrophil cytoplasmic antibodies; NETs, neutrophil extracellular traps; BAFF, B-cell activating factor; APRIL, a proliferation-inducing ligand; PTX3, pentraxin-3; vWF, von Willebrand factor; sCD163, soluble CD163; KIM-1, kidney injury molecule-1; uMCP-1, urinary monocyte chemoattractant protein-1. Each node represents a pathogenetic step; ▸ indicates corresponding clinical biomarker(s).

In Takayasu arteritis, PET/CT provides crucial information on disease extent and activity, identifying metabolically active lesions in patients with apparently normal inflammatory markers—the “imaging-clinical discordance” alluded to above (53, 54). The PETVAS study and subsequent longitudinal analyses have yielded preliminary evidence that persistent PET activity during glucocorticoid therapy identifies patients at higher risk of radiological progression and relapse, supporting the role of PET/CT as a treatment monitoring tool rather than merely a diagnostic one (54, 55) (Figure 2).

For treatment monitoring, the use of PET/CT faces the challenge of glucocorticoid-induced suppression of FDG uptake, which may mask residual active disease (53, 54). Novel radiotracers targeting specific inflammatory pathways—including 68Ga-labeled fibroblast activation protein inhibitor (FAPI) and TSPO ligands directed at activated macrophages—are under investigation and may offer improved specificity for vascular inflammation independent of glucocorticoid therapy (56, 57).

### Vessel wall MRI (VW-MRI): high resolution characterization of arterial wall

High-resolution vessel wall magnetic resonance imaging (VW-MRI) is an emerging technique that directly images the arterial wall, enabling detection of mural enhancement, edema, thickening, and stenosis without ionizing radiation. By using dedicated surface coils, black-blood pulse sequences to suppress intravascular signal, and gadolinium-based contrast agents, VW-MRI can characterize the composition and inflammatory activity of the vessel wall with submillimeter spatial resolution.

In GCA, VW-MRI has demonstrated mural edema and enhancement of the temporal, occipital, and vertebral arteries in active disease, with sensitivities comparable to temporal artery biopsy and the advantage of evaluating bilateral and extracranial vessels simultaneously (58). Multicenter prospective studies have established VW-MRI as a reliable diagnostic alternative to TAB in GCA, particularly in patients where biopsy is technically challenging or contraindicated. Importantly, VW-MRI enhancement resolves with effective treatment, enabling direct visualization of therapeutic response at the level of the vessel wall (58, 59).

In intracranial vasculitis, including primary angiitis of the central nervous system (PACNS), VW-MRI has become the imaging modality of choice for distinguishing inflammatory from non-inflammatory arteriopathies, demonstrating concentric mural enhancement in vasculitis vs. eccentric enhancement in atherosclerosis or vasospasm (60, 61). This has important clinical implications, as PACNS carries significant treatment implications (high-dose immunosuppression) and potential diagnostic overlap with conditions that do not require immunotherapy.

For Takayasu arteritis, VW-MRI enables longitudinal assessment of mural edema (as a marker of active inflammation) and wall thickness progression (as a marker of structural remodeling and fibrosis) (62). Serial VW-MRI has shown that mural enhancement can persist in segments that appear angiographically normal, suggesting subclinical ongoing inflammation even in clinically quiescent patients—a finding with important implications for treatment tapering decisions (62).

### Vascular ultrasound: bedside diagnosis of large vessel vasculitis

Duplex ultrasonography of the temporal arteries—and increasingly of the axillary and subclavian arteries—has gained a prominent role in the rapid diagnosis of GCA. The characteristic “halo sign,” defined as a hypoechoic, non-compressible circumferential thickening of the temporal artery wall, has been validated against temporal artery biopsy in multiple prospective studies (63). Meta-analyses report a pooled sensitivity of 77% and specificity of 96% for temporal artery ultrasound in GCA, with performance optimized by operator training and same-day examination before steroid initiation (64).

The 2018 EULAR recommendations on the use of imaging in LVV explicitly recognized temporal artery ultrasound as the first-line imaging modality in GCA when performed by trained operators, acknowledging that a positive ultrasound may obviate the need for temporal artery biopsy in the appropriate clinical context (65). The TABUL study, a large UK prospective multicenter study, showed that ultrasound had higher sensitivity but lower specificity than temporal artery biopsy, supporting a complementary or sequential diagnostic role for ultrasound in GCA (66).

Recent advances have extended the utility of ultrasound in GCA beyond the temporal arteries to include the axillary and subclavian arteries (for extra-cranial GCA), the carotid arteries, and even the aorta—though aortic ultrasound is constrained by depth and operator dependency. The integration of contrast-enhanced ultrasound may further enhance sensitivity for mural inflammation detection (67). For longitudinal monitoring of GCA disease activity, the OMERACT GCA Ultrasonography Score (OGUS) has been developed as a standardized scoring system enabling reproducible quantification of vascular inflammation across multiple arterial territories (68). A recent multicenter prospective study demonstrated that higher OGUS at diagnosis was associated with increased relapse risk, and that OGUS normalization within the first 3 weeks was independently associated with lower subsequent relapse rate, supporting its role in risk stratification and treatment monitoring (69). For Takayasu arteritis, ultrasound enables evaluation of carotid and subclavian stenoses but is limited for assessment of the thoracic aorta and its distal branches.

### Angiography (CTA) and MR angiography (MRA): anatomical road map

**Figure 3:**
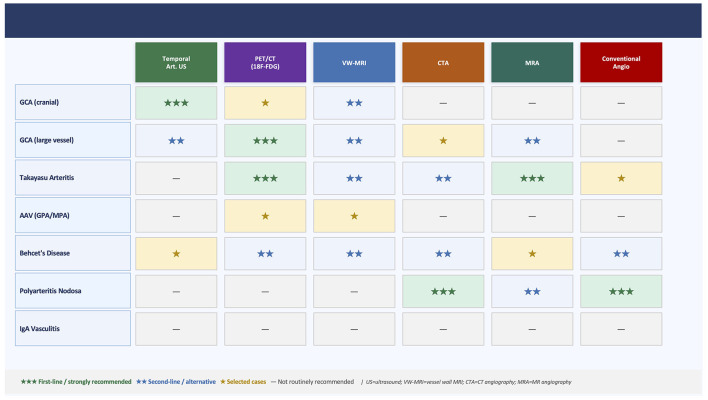
Imaging modalities in vasculitis: diagnostic performance and clinical indications by vessel caliber and disease entity. Matrix summarizing the principal imaging techniques—ultrasound, PET/CT, VW-MRI, CTA/MRA—across the major vasculitic syndromes, with indication of primary diagnostic role, monitoring utility, and key limitations. FDG, fluorodeoxyglucose; SUVmax, maximum standardized uptake value; US, ultrasound; VW-MRI, vessel wall magnetic resonance imaging; CTA, CT angiography; MRA, magnetic resonance angiography.

CT angiography (CTA) and MR angiography (MRA) are complementary lumenal imaging modalities that provide anatomical information on stenoses, occlusions, aneurysms, and dilatations in large and medium-vessel vasculitis. CTA offers superior spatial resolution, shorter acquisition times, and wide availability, making it the preferred modality for urgent clinical assessment and preoperative planning. MRA avoids ionizing radiation and iodinated contrast, making it preferable for longitudinal surveillance in younger patients (as in Takayasu arteritis) and in those with renal impairment (Figure 3).

In Takayasu arteritis, serial CTA or MRA is the standard approach for monitoring anatomical disease progression—detecting new stenoses, assessing the evolution of pre-existing lesions, and planning vascular interventions (22, 58). Current guidelines recommend annual vascular imaging in patients with established TAK, though the optimal modality and frequency remain subjects of debate. The recognized limitation of both CTA and MRA is their inability to directly visualize mural inflammation: a vessel with a long-standing stenosis may appear identical on lumenal imaging whether the stenosis was caused by fibrotic healing or active inflammation.

In medium-vessel vasculitis, particularly polyarteritis nodosa, CTA is the imaging modality of choice for detecting the characteristic microaneurysms of mesenteric and renal arteries (70, 71). These findings, when bilateral and multiple, are highly specific for PAN in the appropriate clinical context, and CTA has largely replaced conventional angiography for diagnostic purposes in this setting.

### Conventional angiography: an underused but irreplaceable tool

Conventional digital subtraction angiography (DSA)—once the uncontested gold standard for vasculitis diagnosis—has progressively been displaced by non-invasive cross-sectional imaging, and is today performed with decreasing frequency in many centers. This trend reflects a combination of factors: the wider availability and continuous technical improvement of CTA and MRA, the perception of DSA as high-risk, and—frankly—the growing reluctance of interventional radiologists to perform diagnostic angiography in the absence of a simultaneous interventional indication. This reluctance, understandable from a workload and risk management perspective, nonetheless carries real diagnostic costs that the rheumatologist should be aware of when advocating for the investigation.

The diagnostic superiority of DSA over non-invasive imaging persists in specific and clinically important scenarios. In Takayasu arteritis, DSA provides the definitive anatomical map of stenoses, occlusions, and collateral circulation before percutaneous transluminal angioplasty (PTA) or surgical bypass, with spatial resolution that no current CTA system can fully replicate for small-caliber branch vessels. In polyarteritis nodosa affecting the renal and mesenteric arteries, the detection of multiple small microaneurysms—the pathological hallmark of PAN—requires the spatial resolution of DSA; while high-quality CTA can detect larger aneurysms, subtle or small microaneurysms (< 3 mm) may be missed. In Behcet's disease with suspected pulmonary artery or peripheral arterial aneurysms, DSA remains essential before any endovascular intervention, given that catheterisation in an acutely inflamed vessel wall carries a specific risk of pseudo-aneurysm formation at the access site—a complication that can be catastrophic if not anticipated.

The rheumatologist's role in this context is not merely to request DSA but to frame the clinical question precisely and to communicate the diagnostic stakes to the interventional radiology team. A targeted DSA of the renal arteries to detect microaneurysms in suspected PAN, performed under optimal conditions in a stable patient, carries a procedural risk that is acceptably low when weighed against the diagnostic benefit of avoiding empirical immunosuppression in an unconfirmed diagnosis. Similarly, DSA of the pulmonary vasculature in suspected Hughes-Stovin syndrome—where pulmonary artery aneurysms carry a risk of life-threatening hemorrhage—provides the anatomical information necessary for endovascular coiling or surgical ligation that no other modality can deliver with equivalent precision.

The development of high-resolution photon-counting CT angiography represents an emerging technological advance that may progressively narrow the diagnostic gap between DSA and non-invasive imaging, particularly for medium-vessel disease. Early clinical data suggest that photon-counting CTA provides improved delineation of small vessel detail and reduced image noise, potentially enabling detection of sub-5 mm aneurysms that were previously DSA-exclusive territory. However, this technology remains limited in availability (72), and DSA should not be prematurely abandoned pending its clinical validation across diverse vasculitis populations.

**Figure 4:**
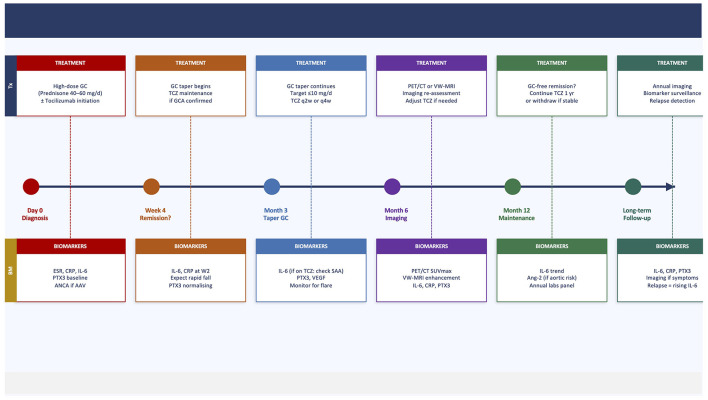
Treatment timeline and biomarker/imaging monitoring in large vessel vasculitis. Schematic representation of the induction and maintenance phases of treatment in GCA and TAK, with indication of recommended biomarker and imaging assessments at each timepoint. TCZ, tocilizumab; RTX, rituximab; GC, glucocorticoids; CRP, C-reactive protein; ESR, erythrocyte sedimentation rate; SAA, serum amyloid A; SUVmax, standardized uptake values; VW-MRI, vessel wall MRI; Ang-2, angiopoietin-2; PTX3, Pentraxin-3; BM, biomarkers; Tx, treatment.

Table 2 provides a comparative overview of the principal imaging modalities used in vasculitis, including their main applications, advantages, and limitations (Figure 4).

**Table 2:** Comparative overview of advanced imaging modaities in vasculitis.

Imaging modality	Vasculitis	Main application	Advantages	Limitations
18F-FDG PET/CT	GCA, Takayasu	Diagnosis, activity, staging	High sensitivity for large vessels; systemic extent; functional activity	Cost; radiation; false positives in atherosclerosis; GC-suppressed uptake
Vessel wall MRI (VW-MRI)	GCA, Takayasu, CNS vasculitis	Mural enhancement, oedema, stenosis	No radiation; excellent wall resolution; treatment response monitoring	Long acquisition; limited availability; motion artifacts
Vascular ultrasound (US)	GCA (temporal, axillary)	Rapid diagnosis; GCA monitoring; US-guided biopsy	Bedside; inexpensive; no radiation; operator-guided biopsy	Operator-dependent; limited for deep vessels
CT angiography (CTA)	Takayasu, PAN, Behcet	Stenoses, aneurysms, dilations	Fast; widely available; high spatial resolution	Radiation; nephrotoxic contrast; limited mural inflammation detail
MR angiography (MRA)	Takayasu, aortic GCA	Lesion extent, longitudinal follow-up	No radiation; excellent luminal anatomy	Limited availability; cost; pacemaker contraindication
Conventional angiography (DSA)	Takayasu, PAN, Behcet	Pre-interventional mapping; microaneurysm detection	Maximum resolution; simultaneous intervention possible	Invasive; radiation; procedural risk; operator-dependent willingness

GCA, giant cell arteritis; TAK, Takayasu arteritis; AAV, ANCA-associated vasculitis, PAN, polyarteritis nodosa; CNS, central nervous system; DSA, digital subtraction angiography; GC, glucocorticoid.

Table 3 summarizes the 2022 ACR/EULAR classification criteria across the major vasculitic syndromes, highlighting the role of biomarkers and imaging as formal classification variables.

**Table 3:** Summary of 2022 ACR/EULAR classification criteria for major vasculitis.

Vasculitis	Key clinical features	Key biomarkers	Key imaging/Histology	Min. score for classification
GCA	Age >=50; headache; jaw claudication; visual loss; scalp tenderness	ESR >=50 mm/h or CRP >=10 mg/L	Positive temporal artery biopsy OR US halo sign OR PET/CT aortic uptake	>=6 points (weighted scoring)
Takayasu arteritis	Age < 60; asymmetric pulses; vascular bruit; limb claudication	ESR or CRP elevated	Typical angiographic or cross-sectional imaging pattern of large vessel stenosis/dilation	>=5 points
GPA	Bloody nasal discharge; nasal polyps; saddle-nose deformity; subglottic stenosis; pulmonary nodules/cavities	PR3-ANCA positive	Granulomatous inflammation on biopsy; pauci-immune GN	>=5 points
MPA	No granulomatous features; rapidly progressive GN; pulmonary fibrosis (ANCA-related)	MPO-ANCA positive	Pauci-immune GN on renal biopsy	>=5 points
EGPA	Asthma; eosinophilia (>=1 × 10^9^/L); nasal polyps; mononeuritis multiplex	ANCA (MPO) in minority; eosinophil count	Eosinophilic granulomatous inflammation; fibrinoid necrosis in small vessels	>=6 points
PAN	Mononeuritis multiplex; testicular pain; myalgia; livedo reticularis; renovascular hypertension	ANCA negative; HBsAg if HBV-PAN	Multiple microaneurysms on renal/mesenteric arteriography; no GN	>=5 points
IgA vasculitis	Palpable purpura (lower limbs); arthritis; abdominal pain; haematuria	Elevated serum IgA; Gd-IgA1 (investigational)	IgA-dominant immune deposits on biopsy (skin or kidney)	>=5 points

GCA, giant cell arteritis, TAK, Takayasu arteritis; GPA, granulomatosis with polyangitis; MPA, microscopic polyangitis; EGPA, eosinophilic GPA; PAN, polyarteritis nodosa; GN, glomerulonephritis; HBV, hepatitis B virus; Gd-IgA1, galactose-deficient IgA1.

## Disease-specific biomarker and imaging approaches

### Giant cell arteritis (GCA)

Giant cell arteritis is the most common primary systemic vasculitis in adults over 50 years, predominantly affecting the cranial branches of the aortic arch and carrying risk of irreversible visual loss and stroke (2). The diagnostic challenge in GCA lies in its protean presentations—from isolated new-onset headache to constitutional syndrome, jaw claudication, aortic aneurysm, or limb ischemia—and in the frequent discordance between clinical symptoms and laboratory inflammation.

The biomarker landscape in GCA is characterized by the central role of IL-6 as a driver of both systemic inflammation and vascular pathology. Serial IL-6 measurements have been proposed as a guide to glucocorticoid tapering, with rising levels preceding clinical relapse (16). PTX3, as detailed above, provides complementary information on local vascular inflammation, and may be particularly useful in patients receiving tocilizumab where standard acute-phase reactants are suppressed (27). Soluble CD163, osteopontin, and VEGF have been studied as markers of macrophage activation and neovascularization within the inflamed arterial wall (73, 74).

The imaging algorithm in GCA has been transformed by the adoption of vascular ultrasound and PET/CT as first-line modalities. In patients presenting with cranial symptoms, same-day temporal artery ultrasound can confirm diagnosis within hours and initiate treatment, with biopsy reserved for equivocal cases (65). In patients with predominantly constitutional features or suspected large-vessel involvement, PET/CT offers comprehensive assessment of aortic and branch vessel involvement. VW-MRI provides the most detailed characterization of mural inflammation and is particularly useful for monitoring treatment response and assessing for aortic complications in the medium term. Nevertheless, important limitations persist. Biomarker studies in GCA are largely observational and constrained by small sample sizes, heterogeneous patient populations, and the lack of standardized definitions of remission and relapse. IL-6 interpretation is confounded by tocilizumab therapy, which paradoxically elevates circulating IL-6 levels while suppressing inflammation. Imaging modalities, though increasingly standardized, still lack universally accepted grading systems for quantifying vascular inflammation, and head-to-head comparisons between ultrasound, PET/CT, and VW-MRI in longitudinal monitoring remain scarce.

### Takayasu arteritis (TAK)

Takayasu arteritis is a granulomatous large-vessel vasculitis predominantly affecting young women, involving the aorta and its major branches with a predilection for the subclavian, carotid, renal, and mesenteric arteries (10). The diagnostic challenge in TAK is the frequent absence of elevated inflammatory markers in patients with active imaging disease—the “imaging-clinical discordance”—which means that reliance on ESR and CRP alone is insufficient for disease activity assessment (23).

Emerging biomarkers in TAK include IL-6 (central to pathogenesis and therapeutic targeting with tocilizumab), PTX3, angiopoietin-2, endothelin-1, and serum soluble HLA class I molecules (22, 75). The TAKT and TOCITAKA trials have validated the efficacy of tocilizumab in refractory TAK, and serial IL-6 and SAA measurements are increasingly used to monitor IL-6 pathway activity during treatment (76, 77). The identification of biomarkers capable of detecting subclinical inflammation before anatomical progression occurs is a critical research priority in TAK.

Imaging in TAK requires assessment of both disease activity (ideally by PET/CT or VW-MRI) and anatomical extent (by CTA or MRA). The EULAR recommendations suggest PET/CT for activity assessment at diagnosis and during follow-up when clinical relapse is suspected, with annual MRA or CTA for structural surveillance (58, 65). The combination of a functional imaging modality demonstrating active wall inflammation with a lumenal imaging modality documenting anatomical progression provides the most complete picture of disease status and informs therapeutic decisions. However, significant limitations remain in the biomarker and imaging landscape of TAK. Most biomarker studies are small, single-center, and retrospective, with inconsistent correlation between inflammatory markers and imaging-defined disease activity—a discordance that reflects the well-recognized dissociation between clinical, laboratory, and imaging remission in TAK. PET/CT availability is limited in many centers, and radiation exposure precludes its unrestricted use in longitudinal monitoring, particularly in young women who represent the majority of TAK patients. VW-MRI, though promising, lacks standardized acquisition protocols and validated scoring systems for routine clinical use.

### ANCA-associated vasculitides (AAV)

ANCA-associated vasculitides—comprising GPA, MPA, and EGPA—are small-vessel vasculitides driven by ANCA-mediated neutrophil and monocyte activation, with a predilection for the kidneys and lower respiratory tract (1, 8). The biomarker landscape in AAV is the most mature among all vasculitic syndromes, with ANCA serology forming the cornerstone of diagnosis and monitoring.

As discussed above, PR3-ANCA and MPO-ANCA have established roles in diagnosis, relapse prediction, and treatment monitoring. The clinical implementation of ANCA-guided rituximab retreatment represents one of the few examples in vasculitis where a biomarker-driven approach has been validated in a randomized controlled trial (15). Beyond ANCA, urinary biomarkers (MCP-1, CD163, TWEAK) hold promise for non-invasive monitoring of renal disease activity, and soluble markers of B-cell and neutrophil activation (BAFF, calprotectin, NETs) are under investigation as complementary tools.

Imaging in AAV is primarily directed at documenting organ-specific damage rather than vascular inflammation *per se*. Chest CT is essential for characterizing pulmonary manifestations (nodules, cavities, ground-glass opacities, and bronchiectasis) and detecting infectious complications in immunocompromised patients. Renal ultrasound assesses kidney size and echogenicity as proxies of chronic damage. The role of PET/CT in AAV is limited compared to LVV, though it may be useful in atypical presentations or when malignancy enters the differential diagnosis (11). Despite these advances, several limitations temper enthusiasm. The majority of biomarker studies in AAV are retrospective, single-center, and underpowered, with significant heterogeneity in patient populations, assay methodologies, and definitions of relapse. Urinary biomarkers and soluble inflammatory markers remain investigational, lacking prospective validation in adequately powered cohorts. Furthermore, the generalizability of findings across PR3-ANCA and MPO-ANCA subtypes—which differ substantially in pathogenesis, organ involvement, and prognosis—is often not addressed in published studies.

### Polyarteritis nodosa (PAN) and other medium-vessel vasculitides

Polyarteritis nodosa is a necrotizing vasculitis of medium-sized muscular arteries without glomerulonephritis or ANCA positivity. The lack of validated biomarkers in PAN is a recognized gap: acute-phase reactants are non-specific, and there are no established serological markers equivalent to ANCA in AAV. Biomarker research in PAN has focused on markers of endothelial injury and neutrophil activation, but no marker has achieved clinical implementation (1).

Imaging remains central to PAN diagnosis and monitoring. CTA or MRA demonstrating multiple renal and mesenteric microaneurysms in a patient with compatible clinical features is highly suggestive, though biopsy of affected tissue (nerve, muscle, and skin) remains the gold standard (71). In HBV-associated PAN, monitoring of viral replication markers (HBV DNA, seroconversion) is an important adjunct to disease activity assessment, as antiviral therapy rather than immunosuppression is the primary treatment modality (78).

### Behçet's disease: the panvasculitis

Behcet's disease (BD) occupies a unique position in the vasculitis classification, and its deliberate inclusion in this review deserves explanation. BD is the only vasculitic syndrome capable of involving vessels of all calibers—from capillaries and venules (mucocutaneous, ocular) to medium arteries (pulmonary, renal, peripheral) and the aorta—earning it the designation of “panvasculitis” and making it the paradigmatic example of systemic vasculitis in its broadest biological sense (79). Its pathogenesis combines features of autoinflammation (neutrophil hyperactivation, IL-1/IL-17/IL-23 axis) with classical autoimmune mechanisms, without a single defining autoantibody, which renders biomarker research in BD particularly challenging.

From a biomarker perspective, BD lacks the serological precision of ANCA in AAV or IL-6 in GCA. Acute-phase reactants (ESR, CRP) are elevated during flares but non-specific. The neutrophil-to-lymphocyte ratio (NLR)—calculated from a standard complete blood count—has emerged as a simple, inexpensive, and independently validated marker of disease activity and venous thrombosis risk in BD (80). Cytokine profiling demonstrates consistent elevation of IL-17A, IL-18, and TNF-alpha during active disease, underpinning the therapeutic rationale for anti-TNF agents (infliximab, adalimumab) in refractory BD, particularly uveitis and vascular disease. Endothelial activation markers—soluble VCAM-1 (sVCAM-1), sICAM-1, and von Willebrand factor—are elevated in vascular BD and may precede overt thrombotic events (79, 81).

A clinically critical and frequently underappreciated aspect of BD is that its vascular thrombosis is fundamentally inflammatory rather than purely coagulation-driven. Venous thrombosis in BD—the most common vascular manifestation—is characterized by intense adherence of the thrombus to the inflamed vessel wall, rendering anticoagulation alone ineffective and potentially dangerous (particularly with concomitant pulmonary artery aneurysms). This has profound implications for biomarker interpretation: coagulation markers such as D-dimer and Factor VIII are elevated but do not guide the same therapeutic response as in non-inflammatory thrombosis; the primary therapeutic target remains immunosuppression (79).

The imaging approach in BD must be tailored to the clinical phenotype. For vascular BD—comprising arterial aneurysms (pulmonary artery aneurysms in Hughes–Stovin syndrome, peripheral and aortic aneurysms) and venous involvement (deep vein thrombosis, Budd-Chiari syndrome, inferior and superior vena cava thrombosis)—CTA or MRA provides anatomical mapping, while DSA is essential before any endovascular repair given the high risk of pseudo-aneurysm formation at the puncture site in active disease. PET/CT has been used in selected cases of vascular BD to assess the metabolic activity of aneurysm walls and to detect occult foci of vascular inflammation, though its role is less established than in GCA and TAK (53, 79). For neuro-Behcet, brain MRI with T2/FLAIR sequences demonstrates characteristic parenchymal lesions with brainstem predilection, and VW-MRI can demonstrate mural enhancement in affected vessels. Cerebrospinal fluid IL-6 is elevated in neuro-Behcet and may assist in distinguishing active inflammatory disease from non-inflammatory mimics (79, 82).

Table 4 summarizes the principal biomarkers and imaging modalities in Behcet's disease, organized by clinical domain.

**Table 4:** Biomarkers and imaging modalities in Behçet's disease by clinical domain.

Domain	Biomarker/Imaging	Clinical value	Notes
Serological—Inflammation	ESR, CRP, SAA	Non-specific but elevated in active disease; baseline reference	Normalize rapidly with colchicine/biologics
Serological—Cytokines	IL-17A, IL-18, TNF-alpha	Elevated in mucocutaneous and vascular flares; IL-17 correlates with uveitis activity	Support for IL-17/IL-23 pathway targeting
Serological—Endothelial	sVCAM-1, sICAM-1, E-selectin	Marker of endothelial activation in vascular Behcet; elevated before thrombosis	Useful in arterial and venous involvement
Serological—Simple indices	Neutrophil-to-Lymphocyte Ratio (NLR)	Independently associated with disease activity and venous thrombosis risk	Inexpensive; easily calculated from CBC
Serological—Coagulation	D-dimer, Factor VIII, von Willebrand factor	Elevated in venous thrombosis; vWF reflects endothelial injury	Behcet thrombosis does NOT require anticoagulation alone—immunosuppression is key
Imaging—Vascular (arterial)	CTA/MRA/DSA	Detect pulmonary artery aneurysms (Hughes-Stovin), aortic aneurysms, peripheral aneurysms	DSA essential before endovascular repair; risk of pseudo-aneurysm at puncture site
Imaging—Vascular (venous)	Doppler US, CTA venography	Deep vein thrombosis, Budd-Chiari, vena cava involvement	Most common vascular manifestation; thrombosis is inflammatory
Imaging—Neuro-Behcet	MRI brain (T2/FLAIR) + VW-MRI	Parenchymal lesions (brainstem predilection); dural sinus thrombosis; mural enhancement	MRI more sensitive than CT; CSF IL-6 elevated in neuro-Behcet
Imaging—Ocular	Fluorescein angiography (FA), OCT	Retinal vasculitis, foveal involvement, macular oedema	Gold standard for uveitis activity; guides anti-VEGF or biologics

NLR, neutrophil-to-lymphocyte ratio; sVCAM-1, soluble vascular cell adhesion molecule-1; CBC, complete blood count; CTA, CT angiography; MRA, magnetic resonance angiography; DSA, digital subtraction angiography; VW-MRI, vessel-wall MRI; FA, fluorescin angiography; OCT, optical coherence tomography; CSF, cerebro spinal fluid.

### IgA vasculitis, cryoglobulinemic vasculitis and other SVV

IgA vasculitis (Henoch-Schönlein purpura in older terminology) is characterized by IgA1-dominant immune complex deposition and classically presents with palpable purpura, arthritis, abdominal pain, and nephritis (1). Serum IgA levels, galactose-deficient IgA1 (Gd-IgA1), and anti-Gd-IgA1 antibodies are under investigation as diagnostic and prognostic biomarkers, with Gd-IgA1 showing particular promise as a marker of renal involvement severity (83). Imaging in IgA vasculitis is largely used to exclude alternative diagnoses rather than to characterize vascular inflammation directly.

Cryoglobulinemic vasculitis, most commonly associated with hepatitis C infection, is characterized by cryoglobulin deposits in small vessel walls (1). The cryoglobulin titer, rheumatoid factor activity, and complement consumption (low C3/C4) serve as disease activity markers, while HCV RNA viral load is a critical therapeutic target biomarker. Novel biomarkers of B-cell activation (BAFF, APRIL) and endothelial damage are being explored in this population.

## Integrated biomarker-imaging strategies: toward precision vasculitis medicine

### The concept of multimodal disease activity assessment

The limitations of individual biomarkers and imaging modalities in capturing the full complexity of vasculitis activity have driven increasing interest in integrated, multimodal approaches to disease assessment. The fundamental premise is that no single marker—whether serological, urinary, or imaging-based—is sufficient to characterize a disease process as heterogeneous and dynamic as vasculitis, and that combining complementary information streams can improve both diagnostic accuracy and treatment monitoring precision.

Composite disease activity indices, such as the Birmingham Vasculitis Activity Score (BVAS) for AAV and the Indian Takayasu Activity Score (ITAS), have incorporated clinical, laboratory, and—more recently—imaging variables into validated scoring frameworks (84, 85). The next generation of activity indices is expected to integrate quantitative biomarker panels and imaging metrics (such as PET SUVmax or VW-MRI enhancement scores) alongside clinical parameters, providing more objective and reproducible measures of disease activity.

### Biomarker-imaging correlations: evidence and clinical implications

Studies correlating serological biomarkers with imaging findings in vasculitis have yielded important insights. In GCA, IL-6 levels correlate modestly with the extent of PET/CT vascular involvement, though this correlation weakens during glucocorticoid therapy (16, 53, 54). PTX3 has been reported to show stronger correlations with large-vessel involvement on PET/CT (86). In TAK, IL-6 levels correlate with new vascular lesions on MRA and with PET uptake at established stenotic sites (22).

An important emerging concept is that of “subclinical residual inflammation”—a state in which imaging demonstrates ongoing vascular wall activity (PET uptake, VW-MRI enhancement) in patients who appear to be in clinical and biochemical remission. This phenomenon, documented in both GCA and TAK, has significant therapeutic implications: patients with subclinical imaging activity may be at higher risk of structural progression and clinical relapse, potentially warranting more conservative glucocorticoid tapering or the continuation of targeted therapies beyond what clinical parameters alone would suggest.

### Practical algorithms for intergrated assessment

Table 5 provides practical recommendations for the integrated use of biomarkers and imaging in common clinical scenarios encountered in vasculitis management, now including Behcet's disease. These recommendations synthesize current evidence with clinical practice experience and are intended to guide—rather than replace—individualized clinical judgment.

**Table 5:** Practical intergrated biomarker-imaging algorithms for common clinical scenarios in vasculitis management.

Clinical scenario	Recommended biomarkers	Recommended imaging	Goal
Suspected GCA (new onset)	IL-6, PTX3, ESR, CRP	Temporal artery US (same day) + PET/CT if systemic	Rapid diagnosis; avoid biopsy when possible
GCA activity monitoring on therapy	IL-6, CRP, VEGF (SAA if on TCZ)	PET/CT or VW-MRI at 6 months	Detect subclinical relapse; guide GC tapering
Suspected AAV with nephritis	ANCA (PR3/MPO), urinary MCP-1, creatinine	Renal ultrasound; VW-MRI if CNS involved	Early diagnosis; target renal biopsy (Berden class)
AAV in remission follow-up	Serial ANCA, galectin-3, BAFF	Not routine; VW-MRI if neurological symptoms	Predict relapse; guide RTX retreatment interval
Suspected Takayasu (young woman)	IL-6, PTX3, ESR	PET/CT or MRA for initial staging	Extent staging; guide therapy initiation
Takayasu on follow-up	IL-6, CRP, angiopoietin-2	Annual MRA or CTA; PET/CT if relapse suspected	Anatomical progression; detect new lesions
Suspected Behcet's disease	IL-17, IL-18, sVCAM-1, NLR	CTA/MRA for vascular Behcet; DSA if aneurysm	Vascular involvement staging; intervention planning
ICI-associated vasculitis	IL-6, ANCA if AAV suspected	Total-body PET/CT; VW-MRI if CNS involved	Diagnosis, staging, ICI continuation decision

GCA, giant cell arteritis; AAV, ANCA-associated vasculitis; TAK, Takayasu arteritis; VW-MRI, vessel-wall MRI; MRA, magnetic resonance angiography; TCZ, tocilizumab; SAA, serum amyloid A; ICI, immune checkpoint inhinitor; DSA, digital subtraction angiography.

Table 6 provides a synthesis of the current evidence on biomarkers and imaging for relapse prediction across the major vasculitic syndromes, with an indication of their predictive performance and clinical applicability.

**Table 6:** Biomarkers and imaging for relapse prediction in vasculitisd.

Biomarker	Vasculitis	Predictive value for relapse	Sensitivity/Specificity	Clinical use
Rising PR3-ANCA titer	GPA	Strong—HR 3–9 vs stable/negative titers in prospective cohorts	Sens ~70%; Spec ~85%	Prompt clinical review; consider RTX retreatment (MAINRITSAN2 model)
Persistent MPO-ANCA positivity	MPA	Moderate—less consistent than PR3; relapse often occurs with stable titers	Sens ~50%; Spec ~75%	Caution: titer alone insufficient; combine with clinical + urine monitoring
Rising IL-6 (on GC taper)	GCA	Moderate—rises 2–4 weeks before clinical flare in GC-treated patients	Sens ~65%; Spec ~80%	Trigger clinical reassessment; consider GC dose increase or TCZ initiation
PTX3 elevation	GCA/TAK	Moderate—correlates with imaging-active disease; useful when CRP normal	Sens ~60%; Spec ~78%	Particularly useful during TCZ therapy (CRP suppressed)
Elevated urinary MCP-1	AAV (renal)	Moderate—rises before creatinine in renal relapse; reflects glomerular macrophage influx	Sens ~72%; Spec ~80%	Non-invasive renal monitoring; may avoid repeat biopsy
Persistent CD19+ B-cells post-RTX	GPA/MPA	Moderate—B-cell reconstitution at 6 months predicts relapse better than ANCA alone	Sens ~60%; Spec ~70%	Guide RTX retreatment timing in ANCA vasculitis maintenance
Elevated BAFF/BLyS	AAV	Emerging—high pre-RTX BAFF associated with shorter relapse-free survival	Insufficient prospective data	Investigational; potentially useful in RTX non-responders
Imaging activity (PET/CT SUVmax)	GCA/TAK	Strong for structural progression—persistent PET uptake in remission predicts new lesions	Sens ~75%; Spec ~82% for radiological progression	Consider at 6 months in LVV; persistent activity may warrant continued therapy

HR, hazard ratio; RTX, rituximab; GC, glucocorticoids; TCZ, tocilizumab; LVV, large-vessel vasculitis; Sens, sensitivity; Spec, specificity.

## Special topics

### Vasculitis in the era of checkpoit inhibitor immunotherapy

The widespread adoption of immune checkpoint inhibitors (ICIs)—anti-PD-1, anti-PD-L1, and anti-CTLA-4 antibodies—as standard cancer therapies has been accompanied by a spectrum of immune-related adverse events (irAEs), among which vasculitis represents an increasingly recognized complication. ICI-associated vasculitis can affect vessels of any caliber, with reported phenotypes including large-vessel vasculitis mimicking GCA or TAK, ANCA-associated vasculitis, and cutaneous vasculitis (87, 88).

The biomarker assessment of ICI-vasculitis is complicated by the underlying malignancy, which can itself elevate inflammatory markers and confound ANCA results. PET/CT—routinely used for oncological staging—may simultaneously reveal vascular FDG uptake consistent with ICI-vasculitis and residual or progressive tumor activity. The diagnostic challenge of distinguishing ICI-vasculitis from other causes of vascular inflammation in the context of cancer therapy requires careful integration of clinical context, biomarker trends, and imaging findings.

From a therapeutic standpoint, ICI-vasculitis generally responds to glucocorticoids, though decisions regarding ICI continuation must balance oncological risk against the severity of the vasculitic complication. Emerging data suggest that biomarker monitoring—particularly IL-6 and ANCA—and serial imaging may guide treatment de-escalation and ICI rechallenge in selected patients.

### COVID-19, post-COVID and vasculitis

SARS-CoV-2 infection has been associated with a spectrum of vasculopathic manifestations, including a hyperinflammatory syndrome in children (multisystem inflammatory syndrome in children, MIS-C) with features overlapping with Kawasaki disease, and a systemic vasculitis-like picture in severely ill adults (89, 90). The molecular mechanisms include direct endothelial infection, complement activation, NET-mediated endothelial injury, and molecular mimicry leading to autoantibody production (91).

*De novo* onset of ANCA vasculitis following SARS-CoV-2 infection and, more rarely, following mRNA vaccination has been reported in case series, though the causal relationship remains uncertain (92, 93). Biomarker monitoring in these patients follows standard AAV protocols, with ANCA serology playing a central diagnostic role. Imaging for COVID-related vasculopathy has focused on CT pulmonary angiography for microthrombi and PET/CT for assessment of multiorgan inflammatory involvement in severe MIS-A (adult MIS).

### Pediatric vasculitis

Vasculitis in children encompasses distinct entities including Kawasaki disease, IgA vasculitis, childhood-onset TAK, and childhood primary angiitis of the CNS (1). The biomarker and imaging approaches in pediatric vasculitis differ from adults in several important respects: echocardiography is central to Kawasaki disease assessment (coronary aneurysms), PET/CT radiation exposure requires particularly careful risk-benefit assessment, and the normal ranges for inflammatory biomarkers differ significantly across pediatric age groups.

NT-proBNP and troponin are emerging biomarkers in Kawasaki disease for detecting coronary artery involvement and myocarditis. In childhood TAK, VW-MRI is increasingly preferred over PET/CT for longitudinal monitoring due to the absence of ionizing radiation, though its availability in pediatric radiology centers remains limited (94, 95). Efforts to standardize pediatric vasculitis biomarker and imaging protocols are ongoing within the Pediatric Rheumatology International Trials Organization (PRINTO) network (96, 97).

## Future directions and unmet needs

Despite the remarkable advances summarized in this review, several critical unmet needs persist in vasculitis biomarker and imaging research. First, the vast majority of biomarker studies are retrospective, single-center, and underpowered, precluding definitive conclusions about clinical utility. Large-scale, prospective, multicenter biomarker validation studies—ideally embedded within clinical trials—are urgently needed to define the clinical performance characteristics of emerging markers across diverse patient populations.

Second, the standardization of imaging protocols remains a significant challenge. PET/CT reporting for vasculitis lacks universally accepted grading scales for vascular FDG uptake, and VW-MRI acquisition parameters vary substantially across centers. The development of standardized imaging protocols and reporting frameworks—analogous to the Liver Imaging Reporting and Data System (LI-RADS) in hepatology (98)—would facilitate multicenter comparisons and clinical implementation.

Third, the translation of multi-omic discoveries into clinically deployable assays requires substantial investment in analytical validation, health economic evaluation, and regulatory approval pathways. Liquid biopsy approaches—measuring circulating cell-free DNA, microRNA, and extracellular vesicles—hold particular promise as minimally invasive, dynamic markers of disease activity and response.

Fourth, the integration of artificial intelligence (AI) and machine learning into biomarker and imaging analysis represents a transformative frontier. AI-assisted analysis of PET/CT and VW-MRI images has demonstrated proof-of-concept capacity to quantify vascular inflammation with precision exceeding human expert assessment, and machine learning models combining biomarker panels, imaging metrics, and clinical variables may achieve superior predictive performance for relapse and organ damage compared to individual approaches (99).

Finally, the development of treat-to-target frameworks in vasculitis—analogous to those established in rheumatoid arthritis—will require validated composite endpoints incorporating biomarker and imaging data alongside clinical outcomes. The EULAR recommendations for GCA and AAV are moving in this direction, but operational definitions of remission that include imaging-defined absence of vascular inflammation are still lacking.

## Conclusions

The management of vasculitis is undergoing a fundamental transformation, driven by a new generation of biomarkers capable of detecting vascular inflammation with greater specificity and sensitivity than traditional acute-phase reactants, and by advanced imaging modalities that enable direct visualization of vessel wall inflammation across the full anatomical range of these diseases.

ANCA serology remains the cornerstone of diagnosis and monitoring in small-vessel AAV, with ANCA-guided therapeutic algorithms now validated in randomized trials. In large-vessel vasculitis, IL-6, PTX3, and endothelial activation markers complement clinical assessment, while PET/CT, VW-MRI, and vascular ultrasound have become indispensable tools for diagnosis, staging, and treatment monitoring. Urinary biomarkers hold promise for non-invasive monitoring of renal vasculitis, and multi-omic platforms are beginning to define molecular signatures of disease activity and therapeutic response.

The emerging paradigm of integrated biomarker-imaging assessment—combining functional and molecular information from multiple sources—promises to enable true precision medicine in vasculitis: individualized treatment decisions informed by a comprehensive, multi-dimensional picture of disease activity. Realizing this vision will require coordinated international efforts in biomarker validation, imaging standardization, and AI-assisted data integration, with the ultimate goal of eliminating preventable organ damage and treatment toxicity in patients with these rare but devastating diseases.
